# Effects of Different Non-Ionic Polysaccharides on the Heat-Induced Gelling Properties of Curdlan

**DOI:** 10.3390/polym16233345

**Published:** 2024-11-29

**Authors:** Guoyan Zhong, Zhaojun Wang, Qiuming Chen, Zhiyong He, Maomao Zeng, Fang Qin, Jie Chen

**Affiliations:** 1 State Key Laboratory of Food Science and Resources, Jiangnan University, Wuxi 214122, China; 6220112121@stu.jiangnan.edu.cn (G.Z.); zhaojun.wang@jiangnan.edu.cn (Z.W.); chenqm@jiangnan.edu.cn (Q.C.); zyhe@jiangnan.edu.cn (Z.H.); mmzeng@jiangnan.edu.cn (M.Z.); 2 School of Food Science and Technology, Jiangnan University, Wuxi 214122, China; 3 Analysis and Testing Center, Jiangnan University, Wuxi 214122, China; qfflast@sina.com

**Keywords:** heat-induced gel, non-ionic polysaccharide, curdlan, pullulan, galactomannans

## Abstract

Curdlan’s application is constrained by high gelation concentration, poor water solubility, and incompatibility with other polysaccharides. To address these limitations, this study investigated the effects of different concentrations (0.05–0.3%) of non-ionic polysaccharides (pullulan (PL), locust bean gum (LBG), guar gum (GG), and konjac gum (KGM)) on the heat-induced gelling properties of curdlan. PL with no branch showed 0.3% enhanced gel hardness. LBG with a small amount of galactose residue and KGM with an acetyl group had similar effects on hardness, while GG with a large amount of galactose residue slightly weakened the mixed gel. The rheological results showed that PL had little effect on curdlan, and LBG and KGM had a positive effect on curdlan unfolding, but 0.3% GG was significantly antagonistic to curdlan. The above results implied that non-ionic polysaccharides without side chains interacted weakly with the curdlan and hardly changed the properties of curdlan. Curdlan unfolding and stable suspension were favored if the structure contained galactose or acetyl side chains that interacted with curdlan through hydrogen bonding. These results suggested an effective way to modify curdlan by strengthening the interaction of curdlan with others and weakening the hydrogen bonding of curdlan to broaden its application in food colloids.

## 1. Introduction

Curdlan is a neutral, linear, unbranched β-1,3-glucan produced by microorganisms. Beyond its biological activities such as immunomodulatory, antiviral, and anti-inflammatory properties, curdlan exhibits unique heat-induced gelling properties, making it useful in food processing for thickening, stability, and texture improvement, as well as in antimicrobial food packaging films [1]. Curdlan forms a firm and elastic gel when heated at 95 °C for 30 min at concentrations above 1% (*w*/*v*). And when the concentration of the curdlan suspension increases from 1% to 2%, the gel strength significantly rises from 270 g/cm^2^ to 750 g/cm^2^ [2].

The mechanism of curdlan gelation is still controversial, but the general view is that it forms right-handed triple helices through strong interchain hydrogen bonds. As the temperature rises above 40 °C, these bonds begin to break, and between 50 °C and 65 °C, curdlan transitions to a single helix with 6/1 helical folds stabilized by newly formed intramolecular hydrogen bonds, forming a thermo-reversible gel. With further heating, hydrophobic interactions begin to occur between the molecules, eventually leading to the formation of a thermo-irreversible gel [3]. Different gel properties can be achieved by adjusting the heating rate, temperature, duration, and curdlan concentration [4,5].

Curdlan has some limitations in practical applications, such as poor water solubility, high gelation concentration, and limited compatibility [6]. Curdlan tends to precipitate or aggregate below 54 °C under acidic or near-neutral conditions, resulting in an uneven distribution within the gel network [7]. This contributes to inconsistent gel strength, where the lower section of the gel is typically stronger than the upper section due to sedimentation during gelation. The concentration required for its gelation (>1%) is relatively high compared to other hydrocolloids, such as low-acyl gellan gum (0.05–0.4%) and high-ester pectin (>0.66%) [8,9]. Furthermore, its gelling temperature (~80 °C) is higher than that of methylcellulose (~52 °C), hydroxypropyl methylcellulose (63–80 °C), and κ-carrageenan (35–65 °C), often resulting in incompatible or phase-separated structures [10,11,12]. Thus, improving the heat-induced gelling properties of curdlan is essential.

Hydrogen bonding plays a crucial role in the gelation and property improvement of polysaccharides, particularly for curdlan [13]. The formation of its unique triple-helix structure and subsequent gel network is mediated by interchain hydrogen bonds, which stabilize the gel matrix and contribute to its mechanical strength and water-holding capacity [14]. Moreover, interactions between curdlan and other polysaccharides often rely on hydrogen bonding to enhance compatibility and gel properties. For instance, polysaccharides with abundant hydroxyl groups or flexible chain conformations can form interchain hydrogen bonds with curdlan, improving gel elasticity, stability, and the overall network density [15,16,17,18]. Therefore, strategies to modify curdlan (e.g., blending it with other polysaccharides) are often aimed at utilizing the hydrogen bonding mechanism to overcome its limitations.

There are several curdlan modification methods, such as physical modification, chemical modification, and enzyme catalysis, among which blending curdlan with other polysaccharides is a promising way to enhance its properties [19]. Polysaccharide blends have been successful in systems such as tara gum–κ-carrageenan, konjac gum–xanthan gum, xanthan gum–sodium alginate, and gellan gum–chitosan composites, enhancing the mechanical strength, stability, and water holding capacity of gels, which are significant for applications in the industry and other fields [20,21,22,23].

Recent studies on curdlan–polysaccharide composite gels have primarily focused on combinations like curdlan–carrageenan, curdlan–gellan gum, curdlan–xanthan gum, and curdlan–konjac gum (under alkaline conditions) [15,16,17,18]. However, there have been no reports on the behavior of curdlan with non-ionic polysaccharides under neutral conditions. Non-ionic polysaccharides are widely used as essential additives in food processing, known for their strong film-forming properties [24]. For example, small amounts of pullulan improved the elasticity of starch gels due to hydrogen bonding interactions with starch molecules, particularly amylose and short-side-chain amylopectin [25]. Guar gum serves as a dispersant in organic systems (containing hydroxyl and carboxyl groups) and as a coagulant in inorganic systems, promoting gel formation [26]. It can be found that curdlan shares characteristics with starch and organic systems containing hydroxyl groups, such as strong hydrogen bonding and water insolubility, which suggests a strong potential for interaction with non-ionic polysaccharides like pullulan and guar gum. Additionally, phase separation tends to occur in most binary systems when polymer concentrations exceed a certain threshold due to thermodynamic incompatibility [27]. Therefore, studying the effects of non-ionic polysaccharides on the heat-induced gelling properties of curdlan is critical.

**Figure 1:**
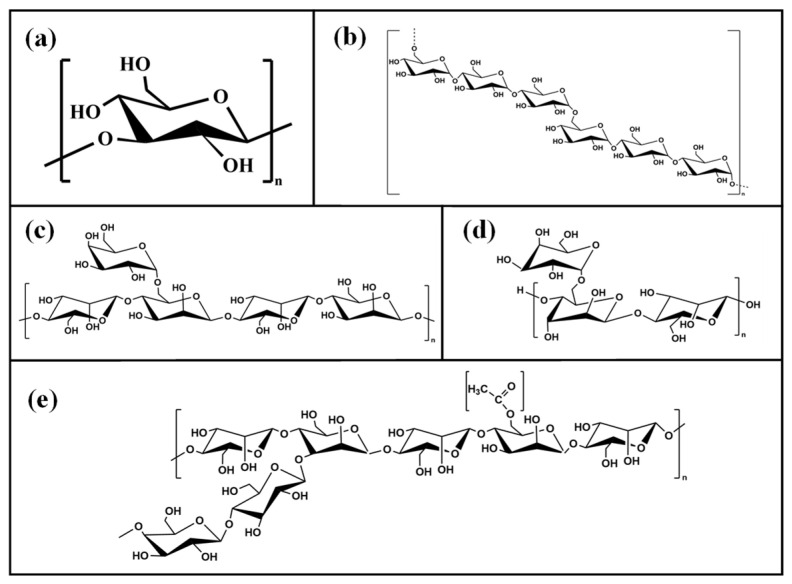
The chemical structures of (**a**) curdlan, (**b**) pullulan, (**c**) locust bean gum, (**d**) guar gum, and (**e**) konjac gum.

Variations in the molecular structure of non-ionic polysaccharides, such as their degree of branching, can lead to distinct hydration behaviors and interactions with other polysaccharides in water [28]. In this study, pullulan (PL), locust bean gum (LBG), guar gum (GG), and konjac gum (KGM) were chosen as representative non-ionic polysaccharides. The chemical structures of all polysaccharides used in this study are shown in Figure 1. PL is a linear, unbranched polysaccharide, while LBG has a mannose backbone with β-D-glucopyranose side chains and a galactose-to-mannose ratio of 1:3.9. GG is a galactomannan with a β-1,4-D-mannose backbone and α-1,6-D-galactose side chains in a galactose-to-mannose ratio of 1:1.8. KGM is composed of glucose and mannose linked by β-1,4-glycosidic bonds, with one acetyl group per 9–20 sugar units. To evaluate the effects of these non-ionic polysaccharides on the heat-induced gelling properties of curdlan, several methods were employed. The rheological properties of composite sols were analyzed, while composite gels were examined by a texture profile analysis (TPA), penetration tests, water holding capacity (WHC), low-field nuclear magnetic resonance (LF-NMR), X-ray diffraction (XRD), and scanning electron microscopy (SEM).

## 2. Materials and Methods

### 2.1. Materials

Curdlan (weight-average molecular weight: 8.6 × 10^5^ g/mol) was supplied by Dongsheng Biotechnology Co., Ltd. (Taixing, China), with a curdlan purity of 96.59%. Locust bean gum (LBG, high purity) was purchased from INCOM END. MAK ITH. IHR. VETIC. A. S. (Mersin, Turkey). Guar gum (GG, high purity) was bought from Beijing Guaran Science and Technology Co., Ltd. (Beijing, China). Pullulan (PL) and konjac gum (KGM), both of analytical grade, were obtained from Shanghai Macklin Biochemical Co., Ltd. (Shanghai, China). All other reagents used were of analytical grade, and deionized water was employed throughout the experiments.

### 2.2. Sample Preparation

A CUR suspension (4 wt%) was prepared by dispersing curdlan powder in deionized water. Other polysaccharides (PL, LBG, GG, and KGM) were prepared at concentrations of 0.1%, 0.2%, and 0.6% (*w*/*w*) using a magnetic stirrer (RT10, IKA, Staufen, Germany). In order to form the CUR-polysaccharide sols, the CUR suspension and the respective polysaccharides were blended at a 1:1 ratio. The mixtures were homogenized at 10,000 rpm for 15 s using a high-speed shearing machine (T18 Basic ULTRA-TURRAX, IKA, Staufen, Germany). The final concentration of CUR was fixed at 2% (*w*/*w*), while the polysaccharides were set at 0.05%, 0.1%, and 0.3% (*w*/*w*) [29]. The curdlan-only suspension was used as the control, while the mixtures were labeled as CUR-PL0.05, CUR-PL0.1, CUR-PL0.3, CUR-LBG0.05, CUR-LBG0.1, CUR-LBG0.3, CUR-GG0.05, CUR-GG0.1, CUR-GG0.3, CUR-KGM0.05, CUR-KGM0.1, and CUR-KGM0.3.

Freshly prepared homogeneous-dispersed polysaccharide sols were used immediately for rheological testing. The remainder was poured into 18 mm × 180 mm glass tubes (15 g per tube) and incubated in a water bath at 100 °C for 30 min. The composite gels were then cooled overnight.

### 2.3. Texture Profile Analysis (TPA)

**Figure 2:**
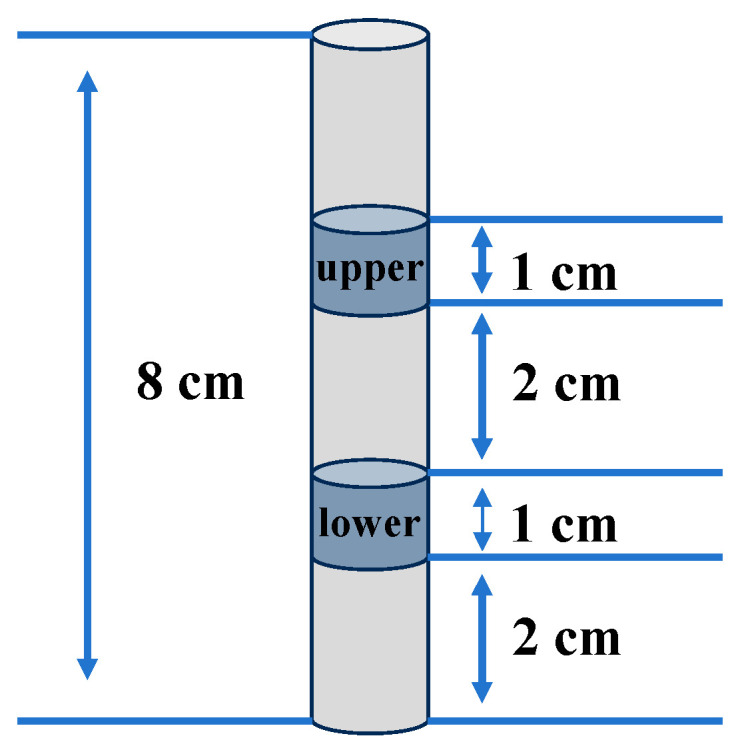
Schematic diagram of the gel sections for testing.

The composite gels were taken out of tubes, and two different sections were cut for testing: the lower section (1 cm) located 2–3 cm from the bottom and the upper section (1 cm) located 5–6 cm from the bottom. As shown in Figure 2, the blue areas represent the gel sections that were taken for testing. TPA were performed using a texture analyzer (TA-XT plus, SMS, Surrey, UK) with a 35 mm diameter cylinder probe (stainless). Samples were compressed at a speed of 2 mm/s with a control force of 5 g and a compression depth of 60%. Each sample was measured 5 times.

### 2.4. Puncture Test

The puncture test used the same sample preparation as for TPA. Testing was performed using a texture analyzer, where a force–time curve (f-t) was recorded to calculate the gel strength, rupture distance, and rupture strength. The probe used was P/5 (stainless), with a test speed of 4.17 mm/s, a puncture depth of 9 mm, and a trigger force of 5 g.

### 2.5. Rheological Properties of Mixed Polysaccharide Sol

The rheological properties were measured using a HAAKE MARS III rheometer (Thermo Scientific, Karlsruhe, Germany). The temperature was controlled by a circulating water bath. The samples were analyzed after being kept for 3 min in a temperature equilibrium and were coated with silicone oil to minimize water loss.

Apparent viscosity: The rheometer adopted a cone plate with a diameter of 35 mm, an angle of 1°, and a gap of 0.052 mm. The apparent viscosity at 25 °C and 60 °C was measured by the variation of shear rates from 0.1 to 100 s^−1^.

Dynamic viscoelasticity and temperature: The rheometer adopted a parallel plate with a diameter of 35 mm and a gap of 1 mm. A strain sweep (0.1 to 100%) was conducted at 25 °C or 60 °C at 1 Hz to determine the linear viscoelastic region (LVR). The temperature ramp began at 25 °C, with a heating rate of 5 °C/min until reaching 95 °C, where it was held for 3 min. Following this, the samples were cooled to room temperature (25 °C) at a cooling rate of 10 °C/min. The testing was conducted at a constant frequency of 1 Hz and a fixed strain of 0.1%. The changes in the storage modulus (G^′^) and the loss modulus (G^″^) with the temperature were recorded and used for a phase shift (tan δ) calculation based on Equation (1).

(1) $$\tan\delta =\frac{G^{\prime \prime }}{G^{\prime }}$$

### 2.6. Water Holding Capacity (WHC)

The WHC was measured by the centrifugation method. All the composite gels were cooled to room temperature before testing. Then, the gels were cut into strips of small pieces (1 cm) with a mold and put into centrifuge tubes filled with cotton at the bottom, weighed as *m*_0_. The weight of the tubes and cotton was referred to as *m*_1_. After the gels were centrifuged at 5000 rpm/min for 15 min (TGL-10B, Shanghai Anting Scientific Instrument Factory, Shanghai, China), the surface moisture was removed with filter paper. The gels were weighted and recorded as m. The WHC was calculated using Equation (2):

(2) $$WHC\left(\%\right)=\frac{m}{m_{0}-m_{1}}\times 100$$

### 2.7. Low-Field Nuclear Magnetic Resonance (LF-NMR)

The transverse relaxation time (T_2_) of the gel samples (1 g) was measured using an LF-NMR analyzer (MesoMR23-060V-1, Niumag Corporation, Shanghai, China). The correction was performed using the oil phase using the Q-FID sequence. The samples were placed in NMR glass tubes and inserted into a 25 mm radio frequency coil. The Carr-Purcell-Meiboom-Gill (CPMG) sequence was used for the tests, with the following parameters: resonance frequency of 21 MHz, spectrum width of 100 kHz, RF delay time of 0.08 ms, time of echoes (T_E_) of 0.6 ms, duration between successive scans (Tw) of 4500 ms, 17,000 echoes (NECH), and 8 scans (NS).

### 2.8. X-Ray Diffraction (XRD)

Composite gels were dehydrated using gradient ethanol (50%, 75%, and 100%) and ground into powder using a mortar, followed by sieving through a 100-mesh screen. X-ray diffraction patterns were collected using an X-ray diffractometer (D2 PHASER, BRUKER AXS, Karlsruhe, Germany) with a scan speed of 5.48°/min over a 2θ range of 5° to 50°, with a step size of 0.05°. The instrument operated with a Cu Ka radiation source at 30 kV and 10 mA.

### 2.9. Scanning Electron Microscopy (SEM)

Five composite gels (control, CUR-PL0.3, CUR-LBG0.3, CUR-GG0.3, and CUR-KGM 0.3) were frozen at –80 °C and freeze-dried for 48 h using a freeze-dryer (SCIENTZ-50F/A, Ningbo Scientzfreeze-drying Equipment Co., Ltd., Ningbo, China). The dried samples were stored in a desiccator at room temperature. To create natural cross-sections, the gels were fractured by gently pulling apart with tweezers. The samples were then mounted on SEM stubs using conductive adhesive and gold-coated under an argon atmosphere. SEM images were captured using a scanning electron microscope (SU8100, Hitachi, Tokyo, Japan) at an accelerating voltage of 3.0 kV.

### 2.10. Statistical Analysis

All experiments were conducted at least in triplicate, with results expressed as mean ± standard deviation using a one-way ANOVA method of Duncan’s test using the SPSS 26.0 software. Special cases will be explained separately. The difference levels of *p* < 0.05 are considered significant differences.

## 3. Results and Discussion

### 3.1. Texture Profile Analysis (TPA) and Water Holding Capacity (WHC)

**Figure 3:**
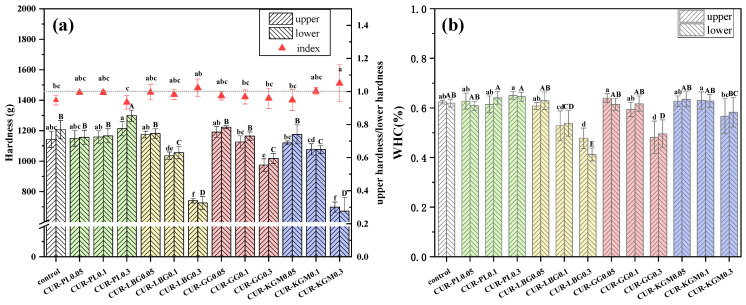
Hardness and WHC of composite gels with varying concentrations of non-ionic polysaccharides. (**a**) Hardness comparison of the upper and lower sections of samples, including the ratio of upper to lower hardness. (**b**) WHC of samples. Different lowercase letters indicate significant differences among the upper sections, and different uppercase letters indicate significant differences among the lower sections (*p* < 0.05).

Hardness is a direct indicator of gel changes. Given that curdlan suspensions tend to settle, hardness was measured at the upper and lower sections of the gels (Figure 3a). The hardness of composite gels significantly increased when the PL concentration reached 0.3%. In contrast, a 0.1% concentration of LBG and KGM significantly reduced the hardness of the composite gels, while a concentration of 0.3% was necessary for GG to achieve a similar reduction. To assess gel uniformity, the hardness ratio between the upper and lower sections was calculated (Figure 3a). A ratio closer to 1 indicates better gel uniformity. The 0.3% PL slightly decreased the uniformity of the composite gel; all concentrations of GG had no effect on the uniformity of the composite gel, while LBG and KGM increased the uniformity of the composite gel, especially 0.3% KGM (*p* < 0.05), which was further confirmed by the wider linear viscoelastic range observed in CUR-KGM0.3 at both 25 °C and 60 °C (Figure S1d,h). However, these observations appeared to contradict the TPA results, as non-ionic polysaccharides that increased or maintained hardness like PL and GG tended to reduce the uniformity of the gels, whereas those that negatively impacted the hardness like LBG and KGM improved the uniformity of the composite gels.

The WHC results were generally similar to the hardness trends, with some slight differences (Figure 3b). CUR-PL0.3 exhibited the highest WHC, and the addition of 0.1% LBG and 0.3% GG significantly reduced WHC. The decrease in WHC could be due to fewer hydrogen bonds within the gel network after the addition of non-ionic polysaccharides, making the three-dimensional network unable to trap free water molecules [30]. The difference with TPA trends was that a concentration of 0.3% KGM did not significantly reduce WHC, only from 61.93% to 58.24%.

When two different polymers in solution are mixed, cooperative interactions can occur, especially in many polysaccharide systems in aqueous solutions. This is well known in mixing xanthan with galactomannans or glucomannans [31,32,33]. Therefore, we can speculate that the interactions between polysaccharides, influenced by their distinct molecular structures, contribute to the observed differences in TPA and WHC (Figure 3). For example, the linear and unbranched structure of PL may lead to limited enhancement of hardness when interacting with curdlan. In contrast, the presence of galactose side chains in LBG and GG, particularly the higher density of side chains in GG, may create stronger interactions with curdlan. Studies have shown that GG is more effective than LBG in enhancing the texture and freeze-thaw stability of rice starch gels [34]. Additionally, the higher galactose content in GG (with a galactose ratio of 1:2) compared to LBG (1:4) results in stronger hydration capacity, which may explain the higher WHC observed in GG.

The gelation of curdlan is also influenced by the structural arrangement of its molecules, the spacing between the triple helices, and the strength of their interactions [14]. When polysaccharides with side chains interact with curdlan, these side chains may occupy potential cross-linking sites, thereby reducing the number of available binding sites for curdlan. This can reduce the ability of curdlan molecules to effectively entangle or cross-link, leading to a decrease in gel hardness. While the structure of KGM likely enabled the strong interactions with curdlan, it only slightly reduced WHC. This is due to its semi-flexible chains and acetyl groups, which allow for helically coiled configurations that create voids within the gel matrix, thus retaining water [35]. Similarly, Liu et al. [36] reported a significant increase in WHC of gel after adding KGM to pork myofibrillar protein.

The polysaccharide hydrogel system is dominated by hydrogen bonding, and the changes in WHC may indicate alternations in hydrogen bonding content within the gel. Polysaccharides contain numerous hydroxyl groups, making them more prone to hydrogen bonding interactions. The improvement in composite gel uniformity is likely due to the high viscosity of the non-ionic polysaccharide, which inhibits particle movement within the system, thereby enhancing the suspension stability of curdlan molecules [37]. Research has reported that adding trehalose enhanced hydrogen bonding interactions with curdlan, significantly increasing gel hardness and stability [38]. Other studies on polysaccharides such as κ-carrageenan, konjac gum, and Mesona chinensis polysaccharide in cassava starch gels have found that these polysaccharides form dense structures with starch through hydrogen bonding, effectively enhancing the dynamic modulus and hardness [39]. Additionally, high curdlan content has been shown to reinforce intermolecular hydrogen bonds with rice starch, thereby improving the gel modulus, quasi-solid behavior, and mechanical properties [40]. These results suggest that varying hydrogen bonding interactions may drive the differences observed in this study. However, the specific interactions between non-ionic polysaccharides and curdlan, as well as their impacts on hardness and WHC, need to be analyzed in later experiments.

### 3.2. Puncture Test

**Figure 4:**
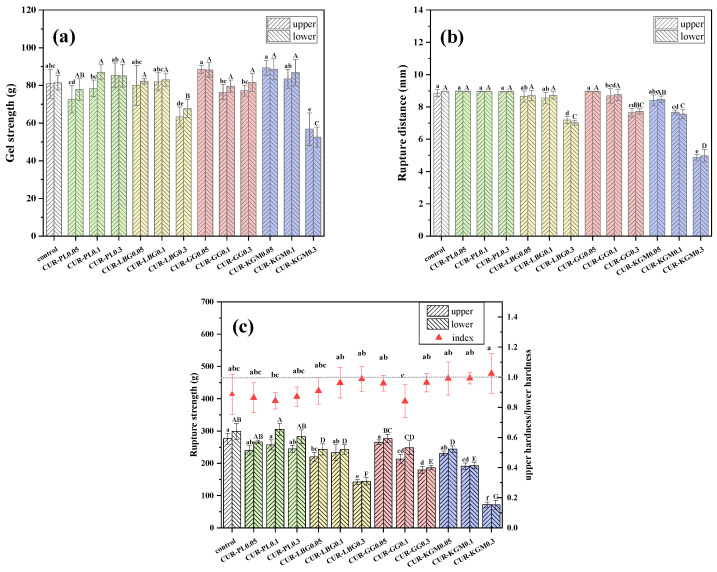
Gel properties of composite gels with varying concentrations of non-ionic polysaccharides. (**a**) Gel strength, (**b**) rupture distance, and (**c**) rupture strength and the ratio index of upper and lower sections. Different lowercase letters indicate significant differences among the upper sections, and different uppercase letters indicate significant differences among the lower sections (*p* < 0.05).

The puncture test was employed to further examine the textural changes in the composite gels. This test measured three key parameters: gel strength, rupture distance, and rupture strength (Figure 4). The overall trend of the gel strength was similar to that of the hardness (Figure 4a). Compared to the control, as the PL concentration increased from 0.05% to 0.3%, the gel strength was first reduced and then increased. The addition of 0.3% LBG and KGM significantly reduced the gel strength (*p* < 0.05), while GG had a negligible effect on reducing gel strength and, in some cases, no reduction at all.

The rupture distance was significantly reduced by the addition of 0.1% KGM, decreasing from 8.47 mm to 7.55 mm (Figure 4b). Moreover, the addition of LBG and GG significantly reduced the rupture distance only when their concentrations reached 0.3%. Gels containing LBG, GG, and KGM were more prone to breaking, while the addition of PL did not change the rupture distance. This observation was consistent with the results of rupture strength (Figure 4c). Gels with added GG, LBG, and KGM showed lower rupture strength compared to the control, with the strength decreasing as the concentration increased. In contrast, the addition of PL did not reduce the rupture strength and even slightly increased it. Figure 4c also illustrates the ratio of the rupture strength between the upper and lower sections of the gel. KGM provided superior stability for the curdlan suspension compared to other non-ionic polysaccharides. Overall, the puncture test results aligned well with those from the TPA test, indicating that there are different interactions and especially hydrogen bonding differences between different non-ionic polysaccharides and curdlan.

### 3.3. Rheological Properties

#### 3.3.1. Apparent Viscosity of Composite Sol

**Figure 5:**
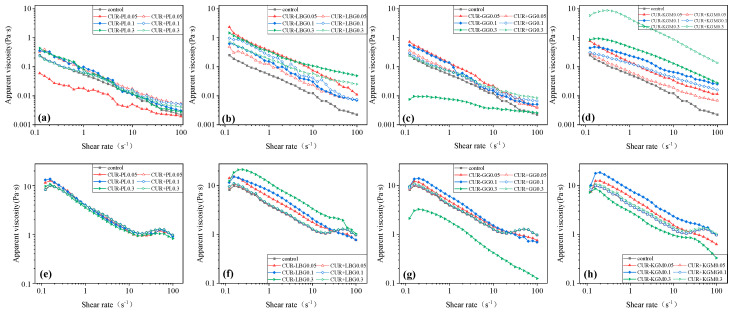
Apparent viscosity of composite sols with different concentrations of non-ionic polysaccharides. (**a**–**d**) represent data at 25 °C, while (**e**–**h**) represent data at 60 °C. Solid symbols indicate actual measured values, and hollow symbols indicate the sum of the viscosities of pure curdlan suspension and the non-ionic polysaccharide solutions at various concentrations.

To investigate the interactions between non-ionic polysaccharides and curdlan, the apparent viscosities of the composite sols were measured at 25 °C (room temperature) and 60 °C (near the first gel transition temperature) (Figure 5). The measured apparent viscosities were compared with the sum of the apparent viscosities of curdlan and the non-ionic polysaccharides at the same concentrations. All samples exhibited shear-thinning behavior with increasing shear rates. This behavior is characteristic of galactomannan solutions under shear, with its extent linked to the density of entanglements and interaction strength, as noted in prior studies [41].

For PL, the apparent viscosity of CUR-PL0.05 was lower than both the theoretical viscosity and the control, while CUR-PL0.1 and CUR-PL0.3 exhibited similar viscosities. At 25 °C, the addition of PL did not significantly affect the viscosity of the composite gel and even exhibited a slight counteractive effect. At 60 °C, the addition of PL had no significant effect on the viscosity of the composite gel. These suggested that PL had weak interactions with the curdlan. The linear structure of PL may be relatively rigid compared to the unbranched curdlan, limiting effective entanglement or cross-linking into complex networks. Additionally, the structure of PL in solution may have competed with water molecules, leading to more intramolecular hydrogen bonds or water-binding interactions rather than strong interactions with curdlan chains. Moreover, curdlan formed a highly cross-linked three-dimensional gel network in water, into which the linear structure of PL may have struggled to incorporate, thus limiting its interactions with curdlan. A previous report indicated that gellan and PL do not form gels at neutral pH [42].

For LBG, the actual viscosity of the composite sol exceeded the theoretical viscosity at both temperatures. At 25 °C, the actual viscosity remained relatively constant across different LBG concentrations, while at 60 °C, the viscosity increased with higher LBG concentrations, indicating a synergistic effect between LBG and curdlan. The lower galactose side-chain content in LBG likely allowed for closer interactions with the curdlan backbone. The steric hindrance from the side chains and these interactions may have helped curdlan molecules unfold more effectively in water, promoting better dispersion and reducing aggregation, which also accounts for the observed improvement in homogeneity [43].

For GG, the actual viscosities at 0.05% and 0.1% concentrations were higher than the theoretical viscosities. But at 0.3%, the viscosity was notably lower than both the theoretical viscosity and the viscosities of lower GG concentrations. At this concentration, GG exhibited a clear antagonistic effect with curdlan at temperatures from room temperature to around 60 °C. This suggests that GG did interact with curdlan to some extent, though less effectively than LBG. This reduced interaction may have resulted from the higher galactose side-chain content in GG, which introduced substantial steric hindrance. While the additional side chains provided more hydrogen bonding sites, they likely reduced interactions between the main chains. Previous studies have noted that a GG film-forming solution exhibits a bean-like morphology due to the relatively dense galactose substitution, which creates steric hindrance. This hindrance impeded the formation of a robust network through intermolecular hydrogen bonding between polymer chains [44].

For KGM, at 25 °C, the actual viscosity increased with the concentration, but at 0.3%, the theoretical viscosities were higher than the actual viscosities, indicating that 0.3% KGM may negatively impact the curdlan system. At 60 °C, the actual viscosity increased with concentration until it dropped below others at 0.3%. These viscosity measurements suggested that KGM likely interacted with curdlan similarly to the CUR-LBG group, enhancing molecular unfolding and, thereby, improving homogeneity. However, the decrease in viscosity at 0.3% may indicate that the composite gel network reached saturation, and further addition of KGM might have led to relaxation or even collapse of the network structure [45].

Overall, non-ionic polysaccharides interacted with curdlan to different extents, resulting in molecular chain cross-linking. Non-ionic polysaccharides without side chains exhibited weaker interactions with curdlan, whereas those with galactose or acetyl side chains were able to interact more effectively with curdlan.

#### 3.3.2. Temperature Ramp

A temperature ramp test was employed to investigate the gelation process of curdlan and its interactions with non-ionic polysaccharides. The control sample exhibited two inflection points, one at approximately 58 °C and another at around 85 °C, consistent with the typical gelation transition temperatures reported for curdlan [14]. Compared to the control, the values of G^′^ and G^″^ for the samples containing non-ionic polysaccharides varied with different polysaccharide types and concentrations, but the overall trend of the curves remained unchanged (Figure S2). This suggests that curdlan continues to dominate the composite gel throughout the gelation process.

The gelation transition temperatures are listed in Table 1. The addition of different non-ionic polysaccharides did not affect the first gel transition temperature but significantly reduced the second gel transition temperature (*p* < 0.05). The addition of non-ionic polysaccharides likely disrupted the original curdlan network by forming new hydrogen bonds that competed with curdlan’s molecular chains. Since the gelation process requires a certain degree of molecular freedom to reconstruct the network, these polysaccharides also lowered the gelation temperature of the system [46]. Studies have shown that LBG at concentrations of 0.4–0.5% reduced both the gelation temperature and gel strength [47]. Other research reported that increased hydrogen bonding contributed to forming a more stable gel network at lower temperatures [48]. Additionally, the presence of more amide groups in pectin enabled denser hydrogen bonding, significantly accelerating the gelation process [49]. These findings suggest that interactions between non-ionic polysaccharides and curdlan were primarily mediated by hydrogen bonding.

### 3.4. Low-Field Nuclear Magnetic Resonance (LF-NMR)

LF-NMR was employed to further analyze the water distribution and states within composite gels, which aided in understanding the interactions between curdlan and non-ionic polysaccharides. The transverse relaxation time T_22_ can indicate the degree of binding between this portion of interstitial water and the components. The T_22_ values and the percentage of water in each component are listed in Table 2. The addition of PL did not affect the T_22_ value. However, significant decreases in T_22_ were observed in CUR-LBG0.1, CUR-GG0.3, and CUR-KGM0.05. The decrease in relaxation time was attributed to the interactions between non-ionic polysaccharides (LBG, GG, and KGM) and curdlan, which contributed to the formation of a three-dimensional network. This resulted in greater constraints on water molecules within the network, reducing their available space for free movement. Additionally, the concentrations at which the relaxation time changed significantly also indicated the extent of interaction with curdlan, consistent with the observations and predictions from the water-holding capacity and rheological experiments.

Regarding the water content in each component, the percentage of strongly bound water remained consistent across all samples, while weakly bound water was undetectable in some samples, and its low content in others offered limited insight. Furthermore, the addition of PL and KGM did not significantly affect the interstitial and free water content in the composite gels. In both the CUR-LBG and CUR-GG groups, a 0.3% addition significantly decreased the amount of interstitial water and increased the free water, consistent with the WHC results (Figure 3b).

The reduction in interstitial water is also related to the gelation mechanism of curdlan. During the thermally irreversible gelation of curdlan, a hydrogen-bond network formed between interstitial water and the triple helix, which then associates into a micellar gel network. The impact of PL was minimal due to its unbranched structure. In contrast, branched polysaccharides such as LBG, GG, and KGM formed hydrogen bonds with curdlan, disrupting the interactions between the original triple helices and interstitial water. The larger molecular volume and steric hindrance introduced by the side chains occupied the pores within the original micelle, reducing the amount of interstitial water. Furthermore, the interference from these polysaccharides hindered the adequate cross-linking of the three-dimensional network of curdlan, affecting the cross-linking and interaction between curdlan molecular chains. This blockage obstructed the close packing necessary for curdlan to form micelles, which explains the observed decrease in hardness, consistent with previous hypotheses. Similar phenomena were reported in xanthan-rich systems [50]. These results suggest that non-ionic polysaccharides, which readily interact with curdlan, can lead to the conversion of interstitial water into free water and potentially alter the structural characteristics of the composite gel, such as causing the micelles to unfold.

### 3.5. X-Ray Diffraction (XRD)

**Figure 6:**
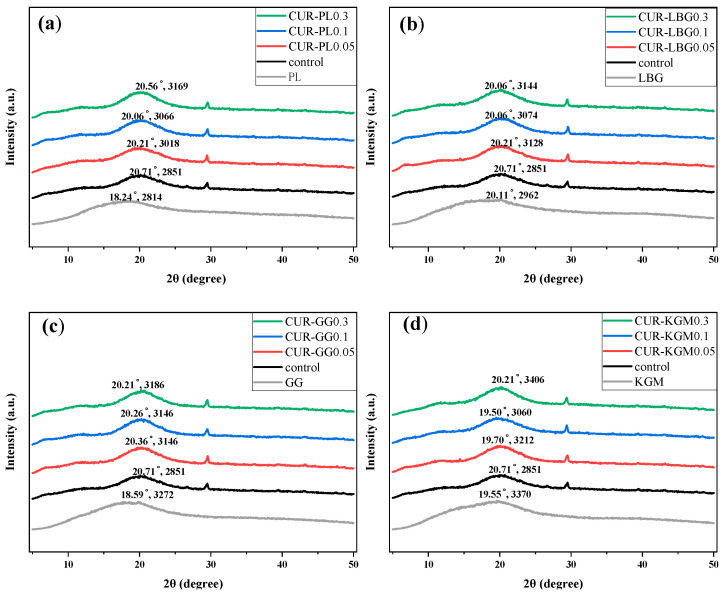
The XRD spectra of composite gels with different concentrations of non-ionic polysaccharides. The values above each curve consist of the angle and the intensity of the diffraction peaks. (**a**) The CUR-PL group. (**b**) The CUR-LBG group. (**c**) The CUR-GG group. (**d**) The CUR-KGM group.

Figure 6 shows XRD spectra of the dehydrated composite gels. The control had low crystallinity, with diffraction peaks appearing at around 6°, 12°, 20°, and 29°, consistent with the report by Tao et al. [51]. The small peak at 6° typically represents the triple-helical conformation of curdlan, while the peaks at 12° and 20° indicate two distinct semi-crystalline regions, reflecting low crystallinity [52]. With the addition of non-ionic polysaccharides, the 2θ values of the characteristic broad peak of the composite gels decreased, falling below the control value of 20.71°, while the diffraction peak intensities increased, all above the control (2851). The addition of these concentrations (0.05–0.3%) did not completely alter the curdlan structure but rather increased the crystallinity, indicating a degree of compatibility between curdlan and the non-ionic polysaccharides at these concentrations. The increase in crystallinity may be attributed to the thermal denaturation of curdlan, where the triple helices dissociate into individual chains. These chains then associate with non-ionic polysaccharides, thereby increasing the crystallinity of the composite gel [53]. Changes in crystallinity could result from the introduction of branched polysaccharides, which might lead to the formation of cross-linked microregions. Over time, crystallization occurs within these aggregated phases [54]. This suggests that non-ionic polysaccharides interact with curdlan, consistent with the apparent viscosity results (Figure 5).

### 3.6. Scanning Electron Microscopy (SEM)

**Figure 7:**
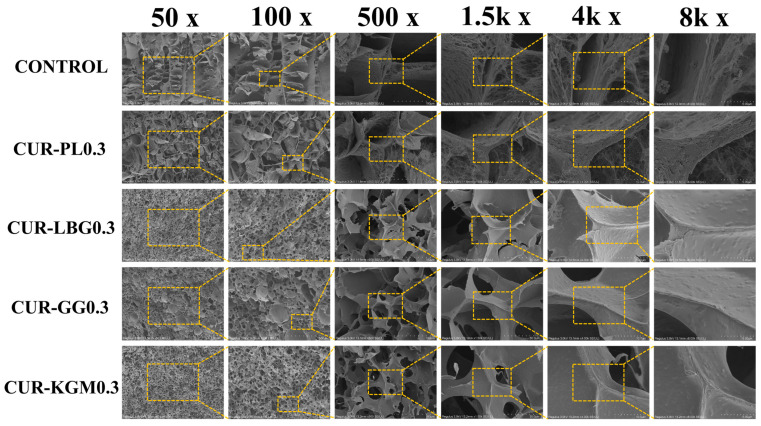
The SEM images of composite gels with different non-ionic concentrations, where yellow boxes highlight the regions of successive magnification.

The microscopic network structure of the composite gels was analyzed at various magnifications (50×, 100×, 500×, 1.5k×, 4k×, and 8k×) (Figure 7). The control sample exhibited a loose network. The introduction of non-ionic polysaccharides likely formed a semi-interpenetrating network, where curdlan established a stable three-dimensional structure, and the polysaccharides intertwined or intercalated into this network [55]. In the CUR-PL 0.3 samples, a relatively loose network was observed, with thinner walls and larger pores compared to the control. However, in the CUR-LBG, CUR-GG, and CUR-KGM groups, smaller gel pores and a more uniform overall structure with denser network walls were noted. This indicates that PL had a minimal impact on the formation of curdlan micelles and molecular cross-linking, whereas other non-ionic polysaccharides, through their branched structures or acetyl groups, interacted more strongly with curdlan. This interaction caused the tightly packed molecular chains necessary for micelle formation to unfold, resulting in a denser gel network [47,50]. This further supports our hypothesis regarding the differences in interactions between these four non-ionic polysaccharides and curdlan, as well as the mechanisms that promote or hinder the unfolding of curdlan molecules.

## 4. Conclusions

In this study, the effects of different types and concentrations of non-ionic polysaccharides on curdlan gels were investigated, focusing on their molecular interactions and the changes in gel properties. The distinct molecular structures of polysaccharides led to varying interesting experimental results. PL, due to its unbranched chain, interacted less with curdlan, thus significantly increasing the hardness at 0.3% but without altering the WHC. Other polysaccharides containing galactose side chains (LBG and GG) and an acetyl group (KGM) formed hydrogen bonds with curdlan, resulting in a decreased gel hardness and WHC with increasing concentration. Among these three, GG interacted the least with curdlan, possibly due to its largest number of side chains and its steric hindrance, leading to a lesser impact on gel hardness and greater WHC than the LBG group. KGM, capable of forming helically voids, maintained a higher WHC compared to the other two polysaccharides. And 0.3% KGM significantly enhanced the uniformity of the gel. Additionally, these interactions promoted a certain degree of unfolding of the curdlan molecules and formed a denser microstructure together, therefore converting the interstitial water in the gel into free water. These results highlighted the significant impact of non-ionic polysaccharide types and concentrations on curdlan gel properties, providing valuable insights for the development of polysaccharide-based gel food products.

## Figures and Tables

**Table 1:** The sol–gel transition temperatures of composite sols with different concentrations of non-ionic polysaccharides. Different letters within each column indicate significant differences (*p* < 0.05).

Sample	First Transition Temperature	Second Transition Temperature
control	58.38 ± 0.53 ^a^	86.24 ± 0.45 ^a^
CUR-PL0.05	57.78 ± 1.13 ^a^	82.78 ± 0.21 ^b^
CUR-PL0.1	57.67 ± 0.73 ^a^	81.65 ± 0.03 ^bc^
CUR-PL0.3	57.50 ± 0.22 ^a^	80.92 ± 0.52 ^bc^
CUR-LBG0.05	58.24 ± 0.25 ^a^	80.92 ± 0.55 ^bc^
CUR-LBG0.1	58.86 ± 0.01 ^a^	80.95 ± 0.53 ^bc^
CUR-LBG0.3	57.21 ± 1.34 ^a^	79.65 ± 2.99 ^c^
CUR-GG0.05	58.11 ± 0.31 ^a^	81.16 ± 2.22 ^bc^
CUR-GG0.1	57.83 ± 1.93 ^a^	81.42 ± 0.79 ^bc^
CUR-GG0.3	57.38 ± 1.87 ^a^	81.63 ± 1.09 ^bc^
CUR-KGM0.05	58.88 ± 1.34 ^a^	82.73 ± 1.98 ^b^
CUR-KGM0.1	57.25 ± 0.60 ^a^	81.32 ± 1.22 ^bc^
CUR-KGM0.3	57.04 ± 0.98 ^a^	82.01 ± 2.60 ^bc^

**Table 2:** The relaxation time T_22_ and relative percentages of different categories of water in CUR-polysaccharide composite gels with different concentrations. Different letters within each column indicate significant differences (*p* < 0.05).

Sample	Transverse Relaxation Time	Relative Percentages of Different Categories of Water			
	T_22_ (ms)	P_20_ (%)	P_21_ (%)	P_22_ (%)	P_23_ (%)
control	264.31 ± 0 ^a^	1.87 ± 0.33 ^a^	/	94.85 ± 1.29 ^ab^	3.28 ± 1.09 ^cd^
CUR-PL0.05	264.31 ± 0 ^a^	1.92 ± 0.07 ^a^	0.12 ± 0.09 ^e^	95.29 ± 0.40 ^ab^	2.71 ± 0.30 ^cd^
CUR-PL0.1	272.85 ± 14.80 ^a^	1.95 ± 0.11 ^a^	/	95.96 ± 0.86 ^ab^	2.09 ± 0.88 ^cd^
CUR-PL0.3	277.13 ± 18.13 ^a^	1.76 ± 0.22 ^a^	0.33 ± 0.21 ^cde^	96.98 ± 0.33 ^a^	0.93 ± 0.32 ^d^
CUR-LBG0.05	256.52 ± 13.49 ^a^	1.90 ± 0.19 ^a^	/	95.29 ± 1.92 ^ab^	2.79 ± 1.80 ^cd^
CUR-LBG0.1	226.74 ± 12.30 ^b^	2.19 ± 0.31 ^a^	0.23 ± 0.11 ^de^	93.79 ± 1.33 ^b^	3.83 ± 1.14 ^c^
CUR-LBG0.3	200.22 ± 0 ^c^	2.27 ± 0.18 ^a^	0.34 ± 0.01 ^bcde^	86.75 ± 2.83 ^d^	10.64 ± 2.66 ^a^
CUR-GG0.05	265.06 ± 24.51 ^a^	1.78 ± 0.17 ^a^	/	94.86 ± 1.34 ^ab^	3.29 ± 1.10 ^cd^
CUR-GG0.1	256.52 ± 13.49 ^a^	1.85 ± 0.32 ^a^	/	95.17 ± 1.53 ^ab^	2.89 ± 1.14 ^cd^
CUR-GG0.3	213.17 ± 11.21 ^bc^	1.76 ± 0.62 ^a^	0.67 ± 0.14 ^abc^	90.60 ± 1.73 ^c^	6.98 ± 1.30 ^b^
CUR-KGM0.05	226.74 ± 12.30 ^b^	1.59 ± 0.44 ^a^	0.56 ± 0.23 ^abcd^	95.08 ± 0.45 ^ab^	2.77 ± 0.43 ^cd^
CUR-KGM0.1	206.69 ± 11.21 ^bc^	1.64 ± 0.30 ^a^	0.68 ± 0.24 ^ab^	94.21 ± 0.72 ^ab^	3.47 ± 0.42 ^cd^
CUR-KGM0.3	213.17 ± 11.21 ^bc^	1.92 ± 0.18 ^a^	0.77 ± 0.27 ^a^	93.23 ± 1.72 ^bc^	4.08 ± 1.69 ^c^

## Data Availability

The original contributions presented in this study are included in the article/Supplementary Material. Further inquiries can be directed to the corresponding author.

## Supplementary Materials

The following supporting information can be downloaded at https://www.mdpi.com/article/10.3390/polym16233345/s1, Figure S1: LVR of composite gels with different non-ionic concentrations; Figure S2: The results of temperature ramp of composite gels with different non-ionic concentrations; Figure S3: The LF-NMR of composite gels with different non-ionic concentrations.
