# Supplementary material for: Effects of Different Non-Ionic Polysaccharides on the Heat-Induced Gelling Properties of Curdlan
Source: Polymers (Basel). 2024 Nov 29;16(23):3345. doi: 10.3390/polym16233345 (PMC11644533; doi:10.3390/polym16233345)
Supplement: Supplementary file 1 [file polymers-16-03345-s001.zip › polymers-3322382-supplementary.pdf]

SUPPORTING INFORMATION

Effects of Different Non-Ionic Polysaccharides on the Heat-Induced Gelling Properties of Curdlan

Guoyan Zhong [1,2], Zhaojun Wang [1,2], Qiuming Chen [1,2], Zhiyong He [1,2], Maomao Zeng [1,2], Fang Qin [3] and Jie Chen [1,2,*]

[1] State Key Laboratory of Food Science and Resources, Jiangnan University, Wuxi 214122, China; 6220112121@stu.jiangnan.edu.cn (G.Z.); zhaojun.wang@jiangnan.edu.cn (Z.W.); chenqm@jiangnan.edu.cn (Q.C.); zyhe@jiangnan.edu.cn (Z.H.); mmzeng@jiangnan.edu.cn (M.Z.)

[2] School of Food Science and Technology, Jiangnan University, Wuxi 214122, China

[3] Analysis and Testing Center, Jiangnan University, Wuxi 214122, China; qfflast@sina.com

* Correspondence: chenjie@jiangnan.edu.cn

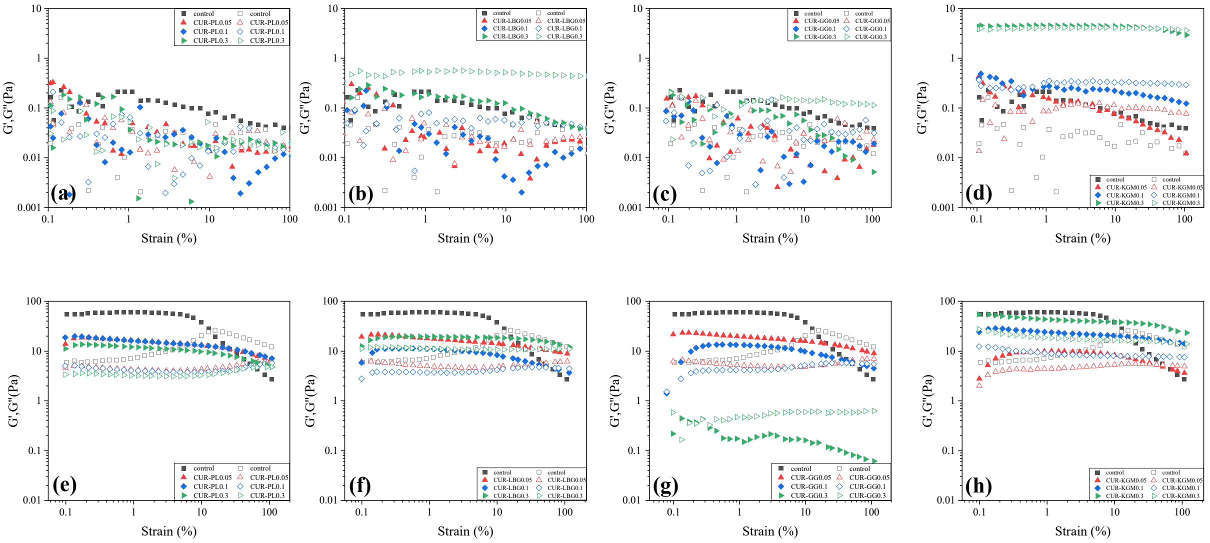

Figure S1. LVR of composite gels with different non-ionic concentrations. (**a**–**d**) show the results at 25 °C, while (**e**–**h**) display the results at 60 °C. Solid symbols represent storage modulus (G'), and hollow symbols represent loss modulus (G'').

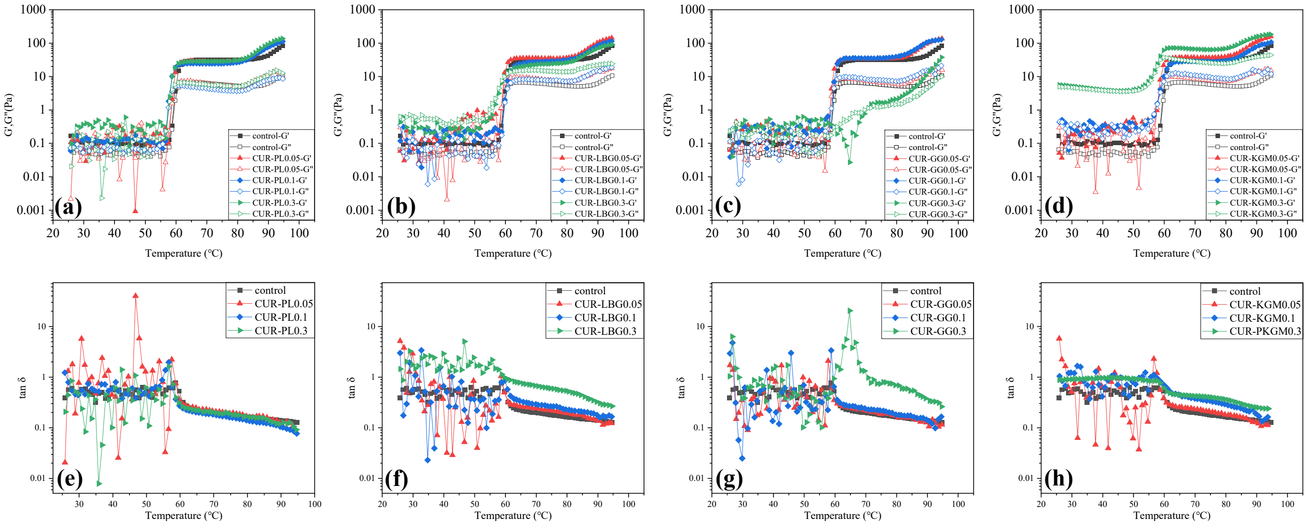

Figure S2. The results of temperature ramp of composite gels with different non-ionic concentrations. (**a**–**d**) The storage modulus (G') and loss modulus (G'') -temperature profiles. (**e**–**h**) The tan δ-temperature profiles.

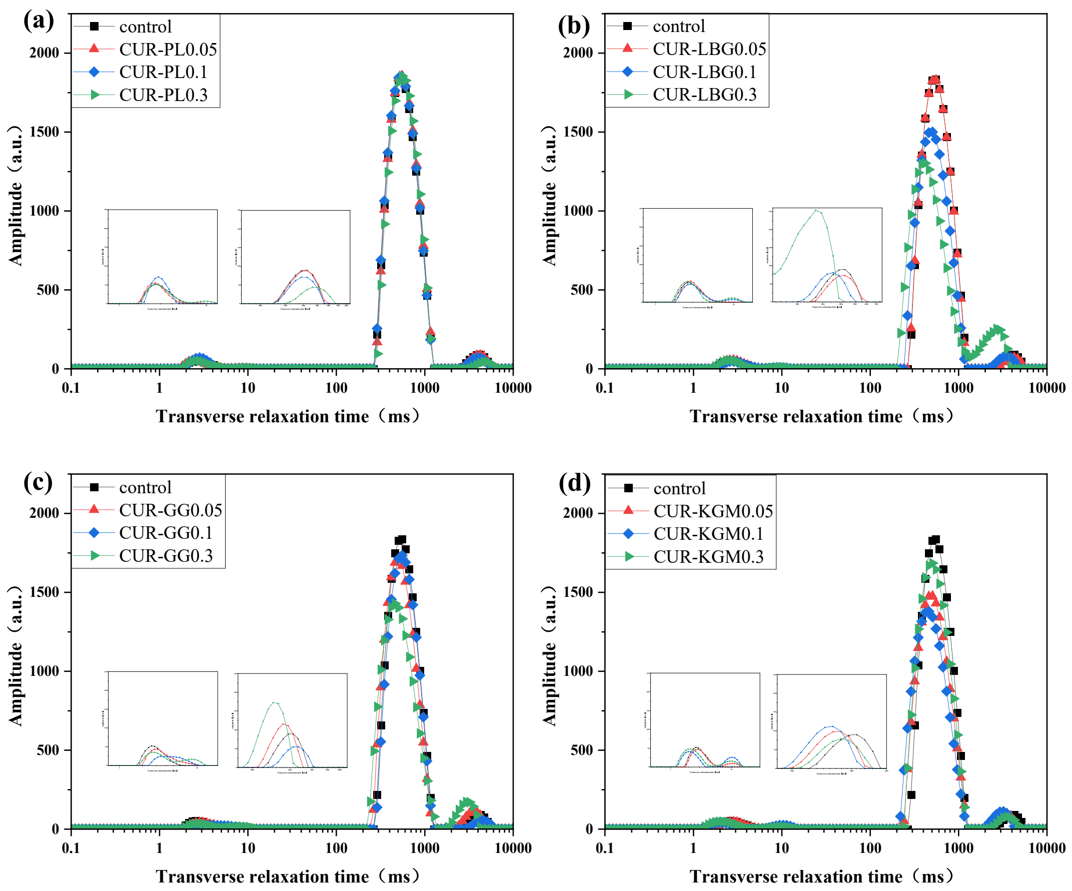

Figure S3. The LF-NMR of composite gels with different non-ionic concentrations. Different letters indicate significant difference (p < 0.05). (a) The CUR-PL group. (b) The CUR-LBG group. (c) The CUR-GG group. (d) The CUR-KGM group.
